# Some Inconsistencies in the Nonlinear Buckling Plate Theories—FSDT, S-FSDT, HSDT

**DOI:** 10.3390/ma14092154

**Published:** 2021-04-23

**Authors:** Zbigniew Kolakowski, Jacek Jankowski

**Affiliations:** Department of Strength of Materials, Faculty of Mechanical Engineering, Lodz University of Technology, Stefanowskiego 1/15, PL-90-924 Lodz, Poland; jacek.jankowski@p.lodz.pl

**Keywords:** nonlinear theories of plate structures, CPT, FSDT, S-FSDT, shear forces, bending and membrane components of transverse forces, transversally inextensible plate, square plate

## Abstract

Bending and membrane components of transverse forces in a fixed square isotropic plate under simultaneous compression and transverse loading were established within the first-order shear deformation theory (FSDT), the simple first-order shear deformation theory (S-FSDT), and the classical plate theory (CPT). Special attention was drawn to the fact that bending components were accompanied by transverse deformations, whereas membrane components were not, i.e., the plate was transversely perfectly rigid. In the FSDT and the S-FSDT, double assumptions concerning transverse deformations in the plate hold. A new formulation of the differential equation of equilibrium with respect to the transverse direction of the plate, using a variational approach, was proposed. For nonlinear problems in the mechanics of thin-walled plates, a range where membrane components should be considered in total transverse forces was determined. It is of particular significance as far as modern composite structures are concerned.

## 1. Introduction

Reissner [1,2] proposed to extend the Timoshenko linear beam theory accounting for a transverse shear effect on the plate theory based on the stress approach 75 years ago. A few years later, Mindlin [3] developed a displacement-based theory, in which it was assumed that transverse shear stresses were constant through the plate thickness. In the case of plates transversely perfectly rigid and at the Mindlin shear correction coefficient *k*^2^ = 5/6, the same stresses were obtained from the Reissner and Mindlin plate theories [4]. Therefore, they are referred to under the common name of the Reissner–Mindlin plate theory. Theoretical considerations and a comparison of both the theories are to be found, for instance, in [5,6,7]. Shear theories of higher orders were discussed in [8,9,10,11,12,13]. In [13], for the functionally graded materials (FGM) plates, a general third-order shear plate theory, in which geometrical nonlinearities were analyzed, was presented. Simplifications of the general theory with 11 general displacements up to 5 displacements for the Reddy third-order theory through the first-order plate theory and to 3 displacements in the classical plate theory (CPT) were proposed. 

In [14,15,16], particular attention was focused on the Reissner boundary effect by an introduction of the rotary potential being a fast-variable solution to the boundary layer. Expressions for the mixed finite element based on the mechanism of a shear locking phenomenon were introduced in [17]. The shear locking phenomenon for the boundary layer in membrane elements was discussed in [6,17,18,19] and was devoted to the finite element method. In the finite element method (FEM), the shear locking phenomenon occurs because a fast-variable solution to the boundary layer cannot be approximated with shape functions [14,20].

Within plate theories covering shearing the first-order shear deformation theory (FSDT), other approaches were developed as well, namely: a two-variable refined plate theory discussed in, for example, [21,22,23,24,25,26,27] and a single-variable refined theory [28].

Endo and Kimura [21] proposed the simple first-order shear deformation theory (S-FSDT). According to this theory, not the angle of rotation in bending but deflection is the primary variable. It imposed simultaneous restrictions on neglecting Reissner boundary effects [2,15]. A number of equations was reduced, and the boundary conditions were altered. In [25,26,27], two independent variables *φ* and *w_s_* were considered, which yielded two differential equations together with boundary conditions. These differential equations are uncoupled in the static analysis, and thus the boundary conditions should be uncoupled as well, however it is impossible. It follows from the fact that a variation in operation of transverse forces is expressed through a difference in virtual displacements. Hence, the fundamental principle of independence of variations is not fulfilled, similarly as it takes place in the FSDT.

A more comprehensive literature survey was presented in [29]. Special attention was paid to membrane components of transverse forces which accompany an appearance of membrane forces in the plate, as predicted by the nonlinear theories: the CPT, the S-FSDT, and the FSDT. It is necessary to apply nonlinear theories to analyze post-buckling equilibrium paths, and in the cases when the plate is subject to loading (e.g., with a transverse load) leading to finite deflections of the plate.

Bending components of transverse forces depend on derivatives of moments, which are accompanied by transverse deformations. These components of forces are linearly dependent on deflection. Membrane components are related to projections of membrane forces on the transverse direction and are nonlinearly dependent on deflection, or more precisely, on deflection raised to the third power. Membrane components of transverse forces do not affect transverse deformations; that is to say, the plate is perfectly rigid with respect to the *z* axis for these components. Thus, in the nonlinear theories—the FSDT and the S-FSDT—double assumptions hold in regard to transverse deformations. For the CPT, an assumption of the plate being perfectly rigid transversely, in which linear bending components, referred to as equivalent Kirchhoff forces and nonlinear membrane components occur, holds. In [29], resultants of these forces were called total equivalent Kirchhoff forces. According to a particular case of Stokes’ theorem, i.e., Green’s theorem, in which the surface integral for the equilibrium equations changes into a plate circumference-oriented integral (i.e., for the boundary conditions), a concept of total equivalent Kirchhoff transverse forces has to be introduced [30].

A transverse shear effect exerts an important influence on the behavior of composite materials characterized by low values of properties referring to transverse shearing [31,32]. 

The achievements of the last decade in the shear deformation theory of thin-walled structures have been discussed in review articles [33,34,35,36,37,38,39,40,41,42], among others.

In the present study, the authors continue their considerations included in [29] in regard to components of transverse forces within the FSDT, the S-FSDT, and the CPT. A detailed analysis is devoted to a square steel plate fixed along all edges and subject to simultaneous compression and transverse loading. Such an example was assumed due to an easy interpretation of the results. In [29], the equations of equilibrium and the boundary conditions following from a variation of the system total energy were derived in detail. The Reissner boundary effect was neglected in the FSDT and the S-FSDT. In the variational approach to the CPT, total equivalent Kirchhoff forces ‘emerge themselves’ in the equations [29]. In the present study, only fundamental equations and their solution for the plate under consideration within the Reissner’s FSDT and the Mindlin’s approach to the S-FSDT (i.e., for two independent functions of displacements along the *z* axis—the total lateral displacement w and the bending deflection φ) and the CPT are discussed.

The three theories presented here, as well as the example, have been known for a long time. Special attention is paid to two components of total transverse forces, i.e., bending components, which are accompanied by transverse deformations, and membrane components, which are not accompanied by transverse shear deformations. Thus, various assumptions in regard to transverse deformations for components of transverse forces are discussed. The above remarks contribute to a disputable nature of this paper. 

## 2. Formulation of the Problem

A nonlinear problem of the distribution of transverse forces in thin-walled plates under simultaneous compression along one direction and the transverse load *q* was investigated. The problem was solved for a square isotropic plate fixed along the whole circumference. The material the plate was made of was assumed to be ruled by Hooke’s law.

Attention was drawn to the theoretical background related to components of transverse forces and a detailed analysis of the distribution of transverse shear forces was carried out. In the analysis, three thin-walled plate theories were considered: the classical plate theory (CPT) (i.e., the Kirchhoff plate theory), the simple first-order shear deformation theory (S-FSDT) in a version of the two-variable refined plate theory, and the Reissner plate theory (FSDT).

The equations of equilibrium and the boundary conditions for the above-mentioned three theories were derived in detail within a variational approach in [29]. In the Appendix A to this study, only the equations necessary to make the paper more articulate are included. A solution for the square isotropic plate, with special attention focused on membrane and bending components of transverse forces, is presented as well. 

Total transverse shear forces (cf. FSDT (A13), S-FSDT (A21), CPT (A28) in the Appendix A, respectively) have two components. Bending components are expressed with the formulas (A11)-FSDT, (A19)-S-FSDT, (A26)-CPT, correspondingly, and they depend on the derivatives of inner moments in the plate. On the other hand, membrane components are expressed with (A12)-FSDT, (A20)-S-FSDT, (A27)-CPT and are related to projections of membrane forces on the direction perpendicular to the central plate plane.

The problem under discussion was solved with two nonlinear equations within the nonlinear theories. One of the them is an equation of inseparability of deformations dependent on the function of forces *F* and the deflection *w*. The second equation is an equation of equilibrium of transverse projections of inner forces on the transverse direction, where we account for the transverse loading *q*, which is written in a simplified version for the three theories under consideration according to (A14), (A22), and (A29) (see the Appendix A) as

(1) $$\int_{0}^{\ell }\int_{0}^{b}[({\hat{Q}}_{x,x}^{\theta }+{\hat{Q}}_{y,y}^{\theta })+({\bar{Q}}_{x,x}^{\theta }+{\bar{Q}}_{y,y}^{\theta })+q]\delta wdxdy=0$$

where the upper index θ=F,S,C refers to the FSDT, the S-FSDT, and the CPT, correspondingly. 

Hence, further on, the following indexes are introduced: C for the CPT; S for the S-FSD, and F for the FSDT, respectively.

In the first round bracket in (1), linear bending components of transverse forces occur, whereas nonlinear membrane components are to be found in the second bracket. According to (A11) for the FSDT, bending components are expressed as a sum of partial derivatives of the bending moment and the torque. In regard to the S-FSDT, it is a similar sum, with such a difference that there is a factor 2 at the torque derivative (cf. (A19) to (A11)). Thus, bending components in the S-FSDT are higher than in the FSDT. For the CPT, formula (A26) is identical as for the S-FSDT. It should be remembered that the expressions for the component moments are, however, different. According to (A61), the respective membrane components of transverse forces ${\bar{Q}}_{x}^{\theta },{\bar{Q}}_{y}^{\theta }$ in (1) for the three theories under consideration are identical, because they depend solely on the variables F,w, which do not affect transverse deformations.

Equation (1) has been expressed in an unusual way in order to draw attention to total transverse forces. When relationships (A13), (A21), and (A28) concerning total transverse forces are considered, Equation (1) can be formulated ultimately as

(2) $$\int_{0}^{\ell }\int_{0}^{b}[{\tilde{Q}}_{x,x}^{\theta }+{\tilde{Q}}_{y,y}^{\theta }+q]\delta wdxdy=0$$

In the above-mentioned two equations, a variation with respect to the defection *w* is included. For the equations derived in [29] in regard to the FSDT and the S-FSDT, components of transverse forces on possible displacements are, respectively

- FSDT

(3) $$\int_{0}^{\ell }\int_{0}^{b}[Q_{x}\delta (w_{,x}-\psi_{x})+Q_{y}\delta (w_{,y}-\psi_{y})]dxdy$$

- S-FSDT

(4) $$\int_{0}^{\ell }\int_{0}^{b}[Q_{x}\delta (w_{,x}-\varphi_{,x})+Q_{y}\delta (w_{,y}-\varphi_{,y})]dxdy$$

when a variation in the plate total potential energy is used.

According to variational principles, Equations (3) and (4) have to be formulated with mutually independent variations of displacements, which is not satisfied in this case. From the authors’ viewpoint, it causes the differential equations to be uncoupled with respect to variations, although the boundary conditions are coupled [29].

Equation (1) for the CPT, when (A24) and two first equations (A10) are accounted for according to (A8) and (A25), leads to one of the commonly known von Karman equations and the second equation is an equation of inseparability of deformations (A9).

(5) $$\int_{0}^{\ell }\int_{0}^{b}[w_{,xxxx}+2w_{,xxyy}+w_{,yyyy}+F_{,yy}w_{,xx}-2F_{,xy}w_{,xy}+F_{,xx}w_{,yy}]\delta wdxdy=0$$

## 3. Results of the Calculations

**Figure 1:**
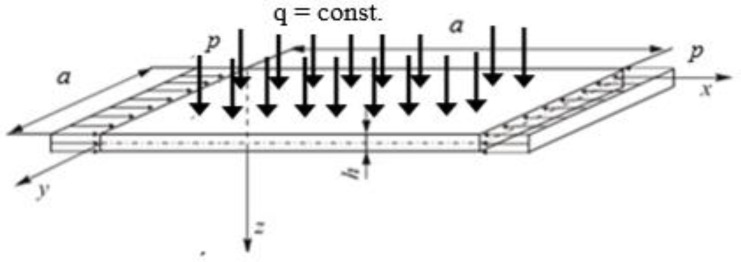
Square plate fixed along all edges and subject to simultaneous compression and uniform transverse load.

A square steel plate (Figure 1) characterized by the geometrical dimensions and material constants equal to: *a* = 100 mm, *h* = 1 mm, E = 200 GPa, ν = 0.3 was analyzed in detail.

The perfect plate was fixed along each edge and uniformly compressed with the stresses p along the *x* axis direction and subject to the transverse load *q*. The boundary conditions for the three theories under analysis are to be found in the Appendix A. 

In the theoretical solution to the nonlinear problem, a simultaneous interaction of the compression *p* and the transverse load *q* was considered so that membrane inner forces could occur, and, consequently, membrane components of transverse forces. In the numerical calculations, two particular cases were analyzed, namely:

- Case A—$p^{C}\neq 0$ and $q^{C}=0$;

- Case B—$p^{C}=0$ and $q^{C}\neq 0$.

It results from the fact that for the compressive load $p^{C}$ in the membrane component (A42) ${\bar{Q}}_{x}^{C}$ along the compression direction for the CPT, the last term is linearly dependent on the deflection *W*, similarly as the bending components. The remaining components of membrane forces are nonlinear with respect to *W*.

In Table 1, results of calculations for both the cases of loads for the assumed values of the dimensionless deflection *W/h* equal to 0.5, 1.0, 1.5, 2.0, respectively, are presented. For the compressive load (Case A), a critical value of load was given and a value of compressive stress, maximal absolute values of bending and membrane components, and total transverse forces were defined for the CPT for the fixed deflection. In regard to the transverse load (Case B), values of the load *q*, maximal absolute values of bending and membrane components, and total transverse forces were determined at the given deflection as in the former case. Concerning the S-FSDT and the FSDT, values of the reduction factor (A45) α=1/(1+η) and values of total transverse forces only for both the cases were given additionally.

Values of bifurcational loads for the FSDT (A58) and the S-FSDT (A44) were the same and inconsiderably lower (by a factor α) than for the CPT (A39). According to (A45), the reduction factor was α = 0.9989 for the assumed data. 

For the case of compression (Case A), values of the maximal absolute bending components of transverse forces ${\left|{\hat{Q}}_{x}^{C}\right|}_{\max}$ and ${\left|{\hat{Q}}_{y}^{C}\right|}_{\max}$ for the CPT, i.e., equivalent Kirchhoff forces, were identical due to two axes of symmetry of the system. Values of the maximal absolute membrane components ${\left|{\bar{Q}}_{x}^{C}\right|}_{\max}$ and ${\left|{\bar{Q}}_{y}^{C}\right|}_{\max}$ differed as a linear relationship between load and deflection occurred in the last term of the first equation of Equations (A42) (for a detailed analysis, see the Appendix A). It caused the total transverse forces (i.e., total equivalent Kirchhoff forces) ${\left|{\tilde{Q}}_{x}^{C}\right|}_{\max}$ and ${\left|{\tilde{Q}}_{y}^{C}\right|}_{\max}$ to be different as well. Maxima of the bending and membrane force components were various for the coordinates *x* and *y*, which resulted in the fact that values of maximal total transverse forces did not sum algebraically. For the values of the dimensionless deflection W/h≤1.5, values of bending components were significantly higher than membrane components. For *W/h* = 2.0, membrane and bending components were almost equal. That rapid increase in membrane components followed from a cubic dependence on the deflection *W*. For the case when only transverse load appeared (Case B), components of transverse forces were equal for the given load due to two axes of symmetry of the system. The remaining results were identical. For *W/h* = 2.0, the value of the transverse load was *q* = 0.54 MPa, which corresponded to uniform loading on the plate surface equal to 5.4 kN. As can be concluded from the above-mentioned considerations, membrane components began to play a significant role starting from deflections equal to the plate thickness. 

For the S-FSDT, conclusions are the same in practice as for the CPT due to a reduction factor close to 1. Differences are to be found in the third or fourth significant figure. Therefore, only maximal values of the absolute forces ${\left|{\tilde{Q}}_{x}^{S}\right|}_{\max}$ and ${\left|{\tilde{Q}}_{y}^{S}\right|}_{\max}$ are listed in Table 1. For the FSDT, the value of critical load was identical to the one for the S-FSDT, as the reduction factors α were the same for both the theories. The bending components ${\left|{\hat{Q}}_{x}^{F}\right|}_{\max}$ and ${\left|{\hat{Q}}_{y}^{F}\right|}_{\max}$ in (A60) were lower than the bending components (A51) for the S-FSDT, because in the second term in the bracket (A60), the factor was equal to 2, whereas in (A51) for the assumed constants, when (A41) was considered, the factor was (3−ν)=2.7. It should be remembered that the membrane components (A61) for the three theories were the same.

Table 1 presents also values of the ratios ${\left|{\bar{Q}}_{x}^{\theta }\right|}_{\max}/{\left|{\tilde{Q}}_{x}^{\theta }\right|}_{\max}$ and ${\left|{\bar{Q}}_{y}^{\theta }\right|}_{\max}/{\left|{\tilde{Q}}_{y}^{\theta }\right|}_{\max}$ (where θ=S,F) to evaluate how membrane components affected total transverse forces. As can be easily noticed, for *W/h* ≥ 0.5 that effect was at least 15%, and for *W/h* = 2.0, it was as high as 50%. It can be said on this basis that for the linear theories S-FSDT and FSDT, membrane components of transverse forces can be neglected for *W/h* < 0.5 (the error was up to approx. 15%). For higher deflections of *W/h*, a nonlinear analysis should be conducted, which enforces the consideration of membrane components of transverse forces.

**Figure 2:**
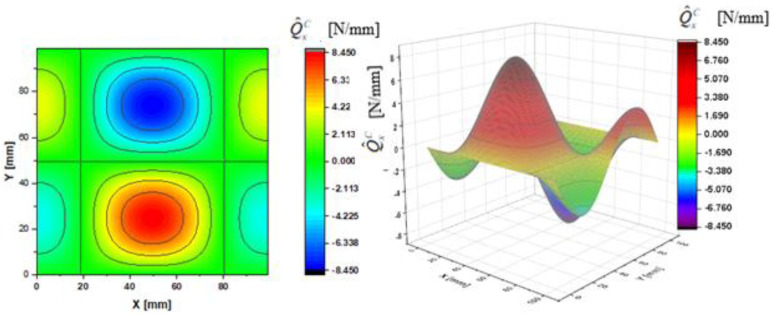
Contour-plane chart of ${\hat{Q}}_{x}^{C}$ for Case A.

**Figure 3:**
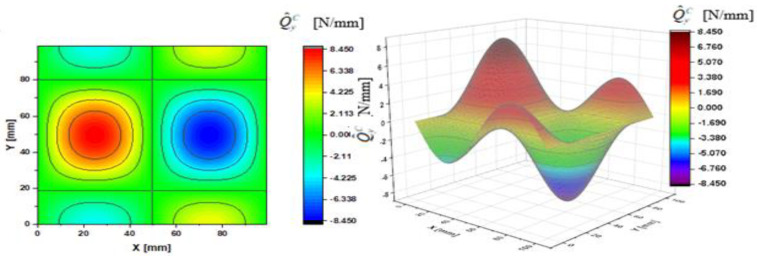
Contour-plane chart of ${\hat{Q}}_{y}^{C}$ for Case A.

**Figure 4:**
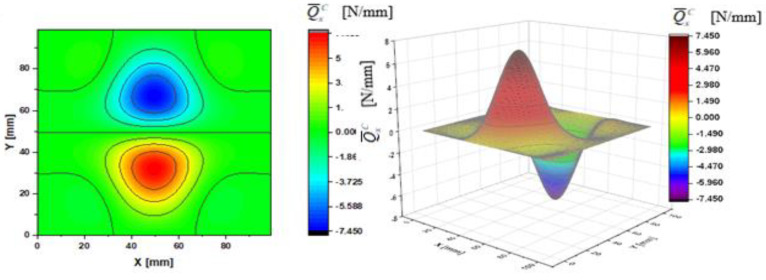
Contour-plane chart of ${\bar{Q}}_{x}^{C}$ for Case A.

**Figure 5:**
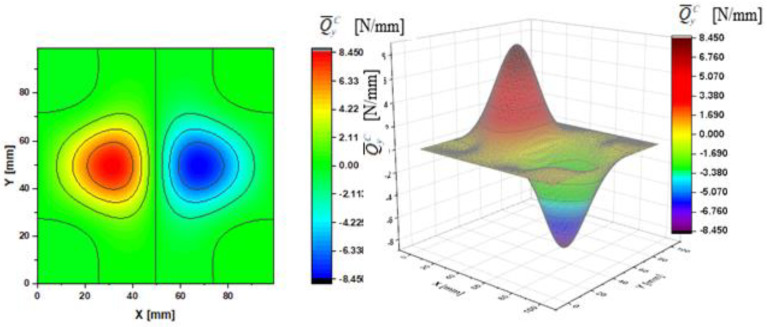
Contour-plane chart of ${\bar{Q}}_{y}^{C}$ for Case A.

**Figure 6:**
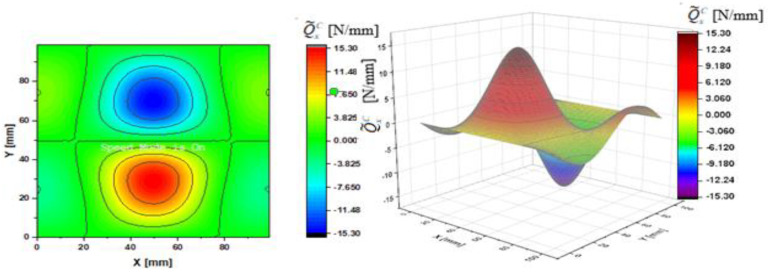
Contour-plane chart of ${\tilde{Q}}_{x}^{C}$ for Case A.

**Figure 7:**
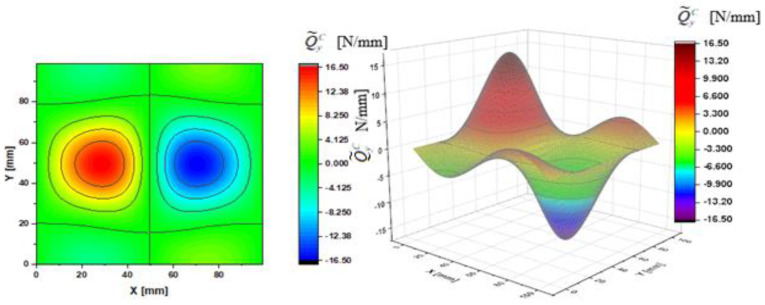
Contour-plane chart of ${\tilde{Q}}_{y}^{C}$ for Case A.

**Figure 8:**
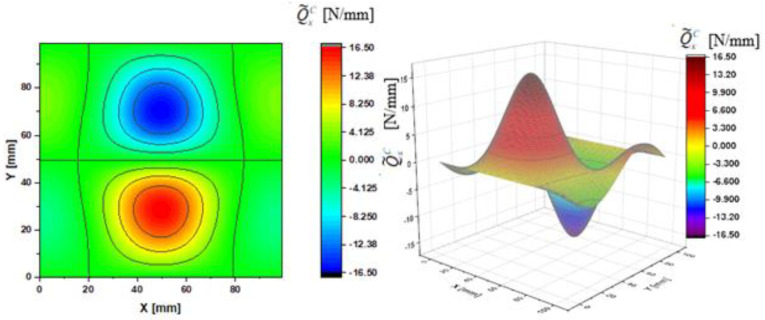
Contour-plane chart of ${\tilde{Q}}_{x}^{C}$ for Case B.

**Figure 9:**
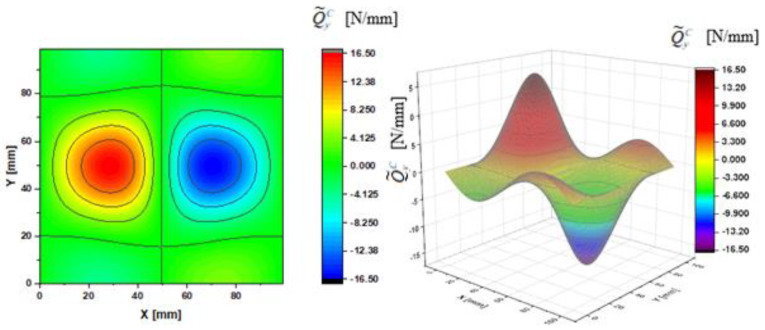
Contour-plane chart of ${\tilde{Q}}_{y}^{C}$ for Case B.

In Figure 2, Figure 3, Figure 4, Figure 5, Figure 6 and Figure 7, contour-plane charts of the transverse forces ${\hat{Q}}_{x}^{C},{\hat{Q}}_{y}^{C},{\bar{Q}}_{x}^{C},{\bar{Q}}_{y}^{C},{\tilde{Q}}_{x}^{C},{\tilde{Q}}_{y}^{C}$ for Case A and the deflection *W/h* = 2.0 are presented for the CPT. It should be remembered that transverse forces are the anti-symmetry forces on the axes of symmetry. It causes the transverse forces ${\hat{Q}}_{x}^{C},{\bar{Q}}_{x}^{C},{\tilde{Q}}_{x}^{C}$ to be anti-symmetrical with respect to the axis y = a/2 *=* 50 mm (see Figure 2, Figure 4, and Figure 6, respectively), and the forces ${\hat{Q}}_{y}^{C},{\bar{Q}}_{y}^{C},{\tilde{Q}}_{y}^{C}$ with respect to the axis x = a/2 *=* 50 mm (see Figure 3, Figure 5, and Figure 7, respectively). In Figure 8 and Figure 9, contour-plane charts for Case B and the deflection *W/h* = 2.0 are depicted also for the CPT. All transverse forces are practically identical for the CPT and the S-FSDT; moreover, they are higher for the S-FSDT than for the FSDT. 

The contour-plane charts for the bending components ${\hat{Q}}_{x}^{C},{\hat{Q}}_{y}^{C}$ (Figure 2 and Figure 3) are identical, which can be easily noticed when we turn in mind one of them by an angle of 90 deg. The charts have two global extrema and four distinct local extrema each. The charts of the membrane components ${\bar{Q}}_{x}^{C},{\bar{Q}}_{y}^{C}$ (Figure 4 and Figure 5) have only two distinct extrema each, however their absolute magnitudes are different. The extreme values ${\bar{Q}}_{x}^{C}$ are lower due to the linear term dependent on deflection and compressive load in contrast to ${\bar{Q}}_{y}^{C}$. The total transverse forces ${\tilde{Q}}_{x}^{C},{\tilde{Q}}_{y}^{C}$ (Figure 6 and Figure 7) arose from a superposition of the charts of components, respectively. Hence, there are two global extrema and four local ones on them like for the bending components. Of course, the relationship ${\tilde{Q}}_{x}^{C}<{\tilde{Q}}_{y}^{C}$ holds for the extrema. For the assumed value of the deflection *W/h* = 2.0, extreme values of the membrane and bending components are almost the same. Thus, the total components are twice as high in practice as the bending components.

In the formula for the membrane component of the force ${\bar{Q}}_{x}^{C}$ (A42), we have a term dependent on the compressive load, which vanishes for the uniform transverse load (Case B). Thus, the charts with the corresponding pairs of the components of transverse forces are of course equal when rotated by an angle of 90 deg (i.e., ${\hat{Q}}_{x}^{C}={\hat{Q}}_{y}^{C}$, ${\bar{Q}}_{x}^{C}={\bar{Q}}_{y}^{C}$, ${\tilde{Q}}_{x}^{C}={\tilde{Q}}_{y}^{C}$). Due to the above-mentioned reasons, only charts for the total transvers forces ${\tilde{Q}}_{x}^{C},{\tilde{Q}}_{y}^{C}$ are shown for Case B (Figure 8 and Figure 9). The extreme values are the same for both forces of course. In the cases of global extrema in Figure 2, Figure 3, Figure 4, Figure 5, Figure 6, Figure 7, Figure 8 and Figure 9, high gradients of transverse forces can be observed.

From the authors’ viewpoint, the total transverse forces ${\tilde{Q}}_{x}^{C},{\tilde{Q}}_{y}^{C}$ are crucial and they should be used in failure criteria, particularly while referring to composite structures.

In [15], Vasiliev proposed to call the version of S-FSDT accounting for the Reissner effect (i.e., the boundary layer) a modern form of the classical plate theory. In the light of the doubts in regard to the FSDT and the S-FSDT presented here, the authors lean to this suggestion, which is reflected in the title of the present study. 

## 4. Conclusions

An influence exerted by bending and membrane components of transverse forces on total transverse forces was analyzed within the following three theories: the CPT, the S-FSDT, and the FSDT. For the S-FSDT and the FSDT, it was shown that bending components were accompanied by transverse deformations, whereas in regard to the membrane components, the plate was perfectly rigid transversely. For both the theories, various assumptions referring to transverse deformations of plates held. Bending transverse components were linearly dependent on the plate deflection, whereas membrane components—nonlinearly. Membrane components played a more and more important role with an increase in deflections above half the thickness of the plate and were higher than membrane components for the deflection corresponding to the doubled thickness of the plate. 

Attention was also paid to a wrong formulation of the variation in operation of transverse forces that consisted in mutually dependent variations of displacements for the first-order shear deformation theory (Reissner–Mindlin plate theory). A modified formulation of the differential equilibrium equation on the transverse direction was proposed. 

In composite materials, transverse shear effects exert a significant influence on structure delamination, which affects considerably integrity and failure of the structure. An effect of membrane components of transverse forces is neglected in composite failure criteria. From the authors’ point of view, transverse components are predominant in the nonlinear problems of delamination. Thus, they should be included in composite failure criteria, e.g., the Hashin failure criterion for 3D, LaRC04(3D), and matrix failure under the additional condition of $\sigma_{33}=0$.

## Figures and Tables

**Table 1:** Values of loads of the square plate and values of absolute maximal components and total transverse forces.

Symbole			Load							
Theory	Symbol	Unit	Case A				Case B			
			W/h							
			0.5	1.0	1.5	2.0	0.5	1.0	1.5	2.0
CPT	$p_{cr}^{C}$	MPa	192.8				0.0			
	$p^{C}$	-	203	236	291	368	0.0			
	$q^{C}$	MPa	0.0				0.075	0.17	0.32	0.54
	${\left|{\hat{Q}}_{x}^{C}\right|}_{\max}$	*N*/mm	2.10	4.20	6.30	8.40	2.10	4.20	6.30	8.40
	${\left|{\hat{Q}}_{y}^{C}\right|}_{\max}$	*N*/mm	2.10	4.20	6.30	8.40	2.10	4.20	6.30	8.40
	${\left|{\bar{Q}}_{x}^{C}\right|}_{\max}$	*N*/mm	0.08	0.72	2.94	7.40	0.13	1.06	3.56	8.44
	${\left|{\bar{Q}}_{y}^{C}\right|}_{\max}$	*N*/mm	0.13	1.06	3.56	8.44	0.13	1.06	3.56	8.44
	${\left|{\tilde{Q}}_{x}^{C}\right|}_{\max}$	*N*/mm	2.06	4.79	8.95	15.3	2.22	5.16	9.63	16.4
	${\left|{\tilde{Q}}_{y}^{C}\right|}_{\max}$	*N*/mm	2.22	5.16	9.63	16.4	2.22	5.16	9.63	16.4
S-FSDT	$p_{cr}^{S}$	MPa	192.6				0.0			
	α	-	0.9989							
	${\left|{\tilde{Q}}_{x}^{S}\right|}_{\max}$	*N*/mm	2.05	4.79	8.95	15.3	2.22	5.16	9.62	16.4
	${\left|{\tilde{Q}}_{y}^{S}\right|}_{\max}$	*N*/mm	2.21	5.16	9.62	16.4	2.22	5.16	9.62	16.4
	${\left|{\bar{Q}}_{x}^{S}\right|}_{\max}/{\left|{\tilde{Q}}_{x}^{S}\right|}_{\max}$	-	0.04	0.15	0.33	0.48	0.06	0.20	0.37	0.51
	${\left|{\bar{Q}}_{y}^{S}\right|}_{\max}/{\left|{\tilde{Q}}_{y}^{S}\right|}_{\max}$	-	0.06	0.20	0.37	0.51	0.06	0.20	0.37	0.51
FSDT	$p_{cr}^{F}$	MPa	192.6				0.0			
	α	-	0.9989							
	${\left|{\tilde{Q}}_{x}^{F}\right|}_{\max}$	*N*/mm	1.67	4.00	7.78	13.7	1.82	4.37	8.45	14.9
	${\left|{\tilde{Q}}_{y}^{F}\right|}_{\max}$	*N*/mm	1.81	4.37	8.45	14.9	1.82	4.37	8.45	14.9
	${\left|{\bar{Q}}_{x}^{F}\right|}_{\max}/{\left|{\tilde{Q}}_{x}^{F}\right|}_{\max}$	-	0.05	0.18	0.38	0.51	0.07	0.24	0.42	0.57
	${\left|{\bar{Q}}_{y}^{F}\right|}_{\max}/{\left|{\tilde{Q}}_{y}^{F}\right|}_{\max}$	-	0.07	0.24	0.42	0.57	0.07	0.24	0.42	0.57

## Data Availability

Data sharing is not applicable to this article.

## Notation

*a*, *h*	geometrical dimensions of the plate
*D*	plate bending stiffness
*E*, *G*	Young’s and Kirchhoff’s modulus
*F*	function of Airy forces
$N_{x},N_{y},N_{xy}$	inner in-plane sectional forces
$M_{x}^{\theta },M_{y}^{\theta },M_{xy}^{\theta }$	inner bending moment-sectional forces
$p_{cr}^{\theta }$, ${\bar{p}}_{cr}^{\theta }$	critical stress, dimensionless critical stress
$q^{\theta }$, ${\bar{q}}^{\theta }$	transverse loading, dimensionless transverse loading
${\bar{Q}}_{x}^{\theta },{\bar{Q}}_{y}^{\theta }$	membrane components of transverse forces
${\hat{Q}}_{x}^{\theta },{\hat{Q}}_{y}^{\theta }$	bending components of transverse forces
${\tilde{Q}}_{x}^{\theta },{\tilde{Q}}_{y}^{\theta }$	total components of transverse forces
*u*, *v*, *w*	components of the plate displacement along the axis *x*, *y*, *z*
$w_{s}$	shear deflection
α	reduction factor
$\beta_{x},\;\beta_{y}$	angles of transverse shear
$\varepsilon_{x}^{},\varepsilon_{y}^{},\gamma_{xy}^{}$	membrane strains
ζ	dimensionless amplitude deflection
η	dimensionless coefficient
θ=F,S,C	upper index referring to the FSDT, the S-FSDT, the CPT
$\kappa_{x},\;\kappa_{y},\;\kappa_{xy}$	bending strains
ν	Poisson’s ratio
φ	function of potential
$\psi_{x}$,$\psi_{y}$	angles of rotation of the transverse normal due to bending

## Appendix A

### Appendix A.1. FSDT, S-FSDT, and CPT-Fundamental Equations

The equations for the three theories: the first order shear deformation plate theory (FSDT), the simple first-order shear deformation theory (S-FSDT), and the classical plate theory (CPT), were derived in [29]. The equations of equilibrium and the boundary conditions were obtained within a variational approach. In this Appendix, only fundamental equations of these theories, supplemented with an additional inclusion of the transverse load *q* in comparison to [29], are presented.

The geometrical relationships for the FSDT were assumed as

(A1) $$\begin{array}{l} \varepsilon_{x}^{}=u_{,x}+\frac{1}{2}w_{,x}^{2}\\ \varepsilon_{y}^{}=v_{,y}+\frac{1}{2}w_{,y}^{2}\\ 2\varepsilon_{xy}^{}=\gamma_{xy}^{}=u_{,y}+v_{,x}+w_{,x}w_{,y} \end{array}$$

and

(A2) $$\kappa_{x}=-\psi_{x,x};\;\kappa_{y}=-\psi_{y,y};\;\kappa_{xy}=-(\psi_{x,y}+\psi_{y,x})$$

where: *u*, *v*, *w*—components of the vector of the plate displacement along the axis *x*, *y*, *z* direction, respectively; $\psi_{x}$, $\psi_{y}$—angles of rotation of the transverse normal due to bending about the axis *x*, *y*, correspondingly; x−y—central plane prior to buckling; moreover, the notations, e.g., $u_{,x}=\partial u/\partial x$, are introduced.

In the transverse shear plate theory (FSDT), it was assumed that full angles of rotation of the normal to the central surface in two planes were as follows [29]:

(A3) $$w_{,x}=\psi_{x}+\beta_{x}\;w_{,y}=\psi_{y}+\beta_{y}$$

where $\beta_{x},\;\beta_{y}$—angles of transverse shear.

The inner sectional forces were written as

(A4) $$\begin{array}{l} N_{x}=\frac{Eh}{1-\nu^{2}}(\varepsilon_{x}+\nu \varepsilon_{y})\\ N_{y}=\frac{Eh}{1-\nu^{2}}(\varepsilon_{y}+\nu \varepsilon_{x})\\ N_{xy}=\frac{Eh}{1-\nu^{2}}\frac{1-\nu }{2}\gamma_{xy} \end{array}$$

(A5) $$\begin{array}{l} M_{x}^{F}=-D(\psi_{x,x}+\nu \psi_{y,y})\\ M_{y}^{F}=-D(\psi_{y,y}+\nu \psi_{x,x})\\ M_{xy}^{F}=-D\frac{1-\nu }{2}(\psi_{x,y}+\psi_{y,x}) \end{array}$$

(A6) $$\begin{array}{l} {\hat{Q}}_{x}^{F}=k^{2}Gh(w_{,x}-\psi_{x})\\ {\hat{Q}}_{y}^{F}=k^{2}Gh(w_{,y}-\psi_{y}) \end{array}$$

where the upper index *^F^* refers to the FSDT.

System of Equation (A1) can be brought to the form

(A7) $$\varepsilon_{x,yy}^{}+\varepsilon_{y,xx}^{}-\gamma_{xy,xy}^{}=w_{,xy}^{2}-w_{,xx}w_{,xy}$$

When relationships (A4) are accounted for and the function of Airy forces *F* defined as

(A8) $$\begin{array}{l} N_{x}=\sigma_{x}h=F_{,yy}\\ N_{y}=\sigma_{y}h=F_{,xx}\\ N_{xy}=\tau_{xy}h=-F_{,xy} \end{array}$$

is introduced, then finally the equation of inseparability/continuity of deformations is obtained [29]:

(A9) $$\nabla \nabla F\equiv F_{,xxxx}+2F_{,xxyy}+F_{,yyyy}=E(w_{,xy}^{2}-w_{,xx}w_{,xy})$$

The above-mentioned equation is linear with respect to *F* and nonlinear with respect to *w*.

#### Appendix A.1.1. FSDT

For the FSDT, the equations of equilibrium within the variational approach are as follows [29]:

(A10) $$\begin{array}{l} \int_{0}^{l}\int_{0}^{b}[N_{x,x}+N_{xy,y}]\delta udxdy=0\\ \int_{0}^{l}\int_{0}^{b}[N_{xy,x}+N_{y,y}]\delta vdxdy=0\\ \int_{0}^{\ell }\int_{0}^{b}[{\hat{Q}}_{x,x}^{F}+{\hat{Q}}_{y,y}^{F}+{\left(N_{x}w_{,x}+N_{xy}w_{,y}\right)}_{,x}+{\left(N_{xy}w_{,x}+N_{y}w_{,y}\right)}_{,y}+q]\delta wdxdy=0\\ \int_{0}^{\ell }\int_{0}^{b}[M_{x,x}^{F}+M_{xy,y}^{F}-{\hat{Q}}_{x}^{F}]\delta \psi_{x}dxdy=0\\ \int_{0}^{\ell }\int_{0}^{b}[M_{xy,x}^{F}+M_{y,y}^{F}-{\hat{Q}}_{y}^{F}]\delta \psi_{y}dxdy=0 \end{array}$$

The first two equations are satisfied identically by the function of forces *F* (A8).

The following relationships result from the last two relationships (A10):

(A11) $$\begin{array}{l} {\hat{Q}}_{x}^{F}=M_{x,x}^{F}+M_{xy,y}^{F}\\ {\hat{Q}}_{y}^{F}=M_{y,y}^{F}+M_{xy,x}^{F} \end{array}$$

These bending components of transverse forces depend on derivatives of inner moments (A5). The angles of transverse shear $\beta_{x},\;\beta_{y}$ in (A3) correspond to bending components only.

In [29], the following membrane components of transverse forces were introduced:

(A12) $$\begin{array}{l} {\bar{Q}}_{x}^{F}=N_{x}w_{,x}+N_{xy}w_{,y}\\ {\bar{Q}}_{y}^{F}=N_{y}w_{,y}+N_{xy}w_{,x} \end{array}$$

The above-mentioned forces are dependent on projections of membrane forces on the transverse direction and do not affect membrane deformations, i.e., membrane components are not accompanied by deformations in contrast to bending components in the FSDT. Thus, we deal with two different assumptions for bending and membrane components.

According to (A11) and (A12), a concept of components of total transverse forces ${\tilde{Q}}_{x}^{F}$ and ${\tilde{Q}}_{y}^{F}$ was introduced. They are expressed as

(A13) $$\begin{array}{l} {\tilde{Q}}_{x}^{F}={\hat{Q}}_{x}^{F}+{\bar{Q}}_{x}^{F}=(M_{x,x}^{F}+M_{xy,y}^{F})+(N_{x}w_{,x}+N_{xy}w_{,y})\\ {\tilde{Q}}_{y}^{F}={\hat{Q}}_{y}^{F}+{\bar{Q}}_{y}^{F}=(M_{y,y}^{F}+M_{xy,x}^{F})+(N_{y}w_{,y}+N_{xy}w_{,x}) \end{array}$$

When (A12) is taken into consideration in the third relation (A10), an equation of equilibrium is obtained:

(A14) $$\int_{0}^{\ell }\int_{0}^{b}[({\hat{Q}}_{x,x}^{F}+{\hat{Q}}_{y,y}^{F})+({\bar{Q}}_{x,x}^{F}+{\bar{Q}}_{y,y}^{F})+q]\delta wdxdy=0$$

As can be easily noticed, membrane and bending components of transverse forces, as well as the load *q*, occur in the above equation.

On the basis of the analysis of relations (A5) and (A6), it can be stated that the bending components of transverse forces ${\hat{Q}}_{x}^{F}$ and ${\hat{Q}}_{y}^{F}$ are linearly dependent on the variables $\psi_{x},\psi_{y},w$. In turn, the analysis of Formulas (A8) and (A9) referring to the membrane components ${\bar{Q}}_{x}^{F}$ and ${\bar{Q}}_{y}^{F}$ points out to nonlinear dependencies on the variables F,w. Hence, Equation (A14) depends on *w* raised to the third power.

#### Appendix A.1.2. S-FSDT

If the angles of rotation $\psi_{x},\;\psi_{y}$ (A2) are expressed with a new function of potential φ(x,y) [29], such that $\varphi_{,x}=\psi_{x}$, $\varphi_{,y}=\psi_{y}$ and, moreover, if for (A3) the following relationships $\beta_{x}=w_{s,x}$, $\beta_{y}=w_{s,y}$ hold, then

(A15) $$w_{,x}=\varphi_{,x}+\beta_{x}=\varphi_{,x}+w_{s,x}\;w_{,y}=\varphi_{,y}+\beta_{y}=\varphi_{,y}+w_{s,y}$$

An introduction of the function of deflection in bending φ causes the Reissner boundary effects to be neglected [15,29], and the shear deflection is equal to $w_{s}=w-\varphi$. Moreover, the number of variables equal to five (i.e., *u*, *v*, *w*, *ψ_x_*, *ψ_y_*) for the FSDT is reduced to four variables *u*, *v*, *w*, *φ* in the case of the S-FSDT [29]; however, the number of boundary conditions for each boundary does not alter. Thus, boundary conditions are coupled for the S-FSDT formulated in such a way.

To attain uncoupled boundary conditions, the Reissner boundary effect should be accounted for. Then, the equations of equilibrium ought to be supplemented with the second-order equation for the rotary potential (the so-called Helmholtz equation) being a fast-variable solution to the boundary layer. This equation increases the system order up to the sixth. Hence, the boundary conditions are uncoupled.

The Reissner boundary effect occurs under special boundary conditions only. In the FEM analysis, there is a mechanism of the shear locking phenomenon, because a fast-variable solution to the boundary layer cannot be approximated with shape functions.

When (A15) is accounted for, inner forces (A5) and (A6) for the S-FSDT are written as

(A16) $$\begin{array}{l} M_{x}^{S}=-D(\varphi_{,xx}+\nu \varphi_{,yy})\\ M_{y}^{S}=-D(\varphi_{,yy}+\nu \varphi_{,xx})\\ M_{xy}^{S}=-D(1-\nu )\varphi_{,xy} \end{array}$$

(A17) $$\begin{array}{l} {\hat{Q}}_{x}^{S}=k^{2}Gh(w_{,x}-\varphi_{,x})\\ {\hat{Q}}_{y}^{S}=k^{2}Gh(w_{,y}-\varphi_{,y}) \end{array}$$

and the equations of equilibrium [29], when the load *q* is taken into consideration, are

(A18) $$\begin{array}{l} \int_{0}^{\ell }\int_{0}^{b}[{\hat{Q}}_{x,x}^{S}+{\hat{Q}}_{y,y}^{S}+{\left(N_{x}w_{,x}+N_{xy}w_{,y}\right)}_{,x}+{\left(N_{xy}w_{,x}+N_{y}w_{,y}\right)}_{,y}+q]\delta wdxdy=0\\ \int_{0}^{l}\int_{0}^{b}[M_{x,xx}^{S}+2M_{xy,xy}^{S}+M_{y,yy}^{S}-{\hat{Q}}_{x,x}^{S}-{\hat{Q}}_{y,y}^{S}]\delta \varphi dxdy=0 \end{array}$$

where the upper index *^S^* denotes the S-FSDT.

System of Equations (A18) should be supplemented by Equation (A9).

The bending components of transverse forces dependent on the variable φ are expressed with the relationships

(A19) $$\begin{array}{l} {\hat{Q}}_{x}^{S}=M_{x,x}^{S}+2M_{xy,y}^{S}\\ {\hat{Q}}_{y}^{S}=M_{y,y}^{S}+2M_{xy,x}^{S} \end{array}$$

whereas the membrane components of transverse forces dependent on the variables *F*, *w* take the form

(A20) $$\begin{array}{l} {\bar{Q}}_{x}^{S}=N_{x}w_{,x}+N_{xy}w_{,y}\\ {\bar{Q}}_{y}^{S}=N_{y}w_{,y}+N_{xy}w_{,x} \end{array}$$

By analogy to (A13), the components of total transverse forces ${\tilde{Q}}_{x}^{S}$ and ${\tilde{Q}}_{y}^{S}$ for the S-FSDT are expressed as

(A21) $$\begin{array}{l} {\tilde{Q}}_{x}^{S}={\hat{Q}}_{x}^{S}+{\bar{Q}}_{x}^{S}=(M_{x,x}^{S}+2M_{xy,xy}^{S})+(N_{x}w_{,x}+N_{xy}w_{,y})\\ {\tilde{Q}}_{y}^{S}={\hat{Q}}_{y}^{S}+{\bar{Q}}_{y}^{S}=(M_{y,y}^{S}+2M_{xy,y}^{S})+(N_{y}w_{,y}+N_{xy}w_{,x}) \end{array}$$

Comparing the formulas for the bending components of transverse forces (A11) and (A19), one can easily see that we have a factor 2 at the derivative of torque $M_{xy}$ for the S-FSDT, which is 1 in the FSDT. Formulas (A12) and (A20) are identical for the membrane components of transverse forces.

When (A20) is taken into account, the first Equation (A18) takes the form

(A22) $$\int_{0}^{\ell }\int_{0}^{b}[({\hat{Q}}_{x,x}^{S}+{\hat{Q}}_{y,y}^{S})+({\bar{Q}}_{x,x}^{S}+{\bar{Q}}_{y,y}^{S})+q]\delta wdxdy=0$$

The above equation has the same structure as (A14). It should be remembered that bending components are expressed with various formulas (cf. (A11) and (A19)).

Analogously to the FSDT, the bending components of transverse forces ${\hat{Q}}_{x}^{S}$ and ${\hat{Q}}_{y}^{S}$ are linearly dependent on the variable φ, and thus *w* as well, whereas the membrane components ${\bar{Q}}_{x}^{S}$ and ${\bar{Q}}_{y}^{S}$ are nonlinearly dependent on the variables F,w.

#### Appendix A.1.3. CPT

Transverse forces are disregarded in the classical plate theory (CPT) (A6) and it should be additionally assumed that $\beta_{x}=\beta_{y}=0$ in (A3), which leads to the equality

(A23) $$\begin{array}{l} w_{,x}=\psi_{x}\\ w_{,y}=\psi_{y} \end{array}$$

When (A23) is considered in (A5), then for the CPT,

(A24) $$\begin{array}{l} M_{x}^{C}=-D(w_{,xx}+\nu w_{,yy})\\ M_{y}^{C}=-D(w_{,yy}+\nu w_{,xx})\\ M_{xy}^{C}=-D(1-\nu )w_{,xy} \end{array}$$

where the upper index *^C^* refers to the CPT.

When the above relations are included, the equation of equilibrium takes the form [29]

(A25) $$\int_{0}^{\ell }\int_{0}^{b}[M_{x,xx}^{C}+2M_{xy,xy}^{C}+M_{y,yy}^{C}+{\left(N_{x}w_{,x}+N_{xy}w_{,y}\right)}_{,x}+{\left(N_{xy}w_{,x}+N_{y}w_{,y}\right)}_{,y}+q]\delta wdxdy=0$$

The second equation is an inseparability Equation (A9).

In the history of the CPT, equivalent Kirchhoff transverse forces were defined as

(A26) $$\begin{array}{l} {\hat{Q}}_{x}^{C}=M_{x,x}^{C}+2M_{xy,y}^{C}\\ {\hat{Q}}_{y}^{C}=M_{y,y}^{C}+2M_{xy,x}^{C} \end{array}$$

These are bending components of transverse forces and their structure is analogous to (A19). Like for the FSDT and S-FSDT, the membrane components of transverse forces are assumed as

(A27) $$\begin{array}{l} {\bar{Q}}_{x}^{C}=N_{x}w_{,x}+N_{xy}w_{,y}\\ {\bar{Q}}_{y}^{C}=N_{y}w_{,y}+N_{xy}w_{,x} \end{array}$$

Thus, the above-mentioned components of transverse forces are the membrane components of Kirchhoff forces and they have an identical structure as for the FSDT and the S-FSDT.

Considering (A26) and (A27), the total equivalent Kirchhoff transverse forces ${\tilde{Q}}_{x}^{C}$ and ${\tilde{Q}}_{y}^{C}$ for the CPT are expressed in the following form:

(A28) $$\begin{array}{l} {\tilde{Q}}_{x}^{C}={\hat{Q}}_{x}^{C}+{\bar{Q}}_{x}^{C}=(M_{x,x}^{C}+2M_{xy,y}^{C})+(N_{x}w_{,x}+N_{xy}w_{,y})\\ {\tilde{Q}}_{y}^{C}={\hat{Q}}_{y}^{C}+{\bar{Q}}_{y}^{C}=(M_{y,y}^{C}+2M_{xy,x}^{C})+(N_{y}w_{,y}+N_{xy}w_{,x}) \end{array}$$

When (A28) is accounted for in (A25), the equation of equilibrium is written as

(A29) $$\int_{0}^{\ell }\int_{0}^{b}[({\hat{Q}}_{x,x}^{C}+{\hat{Q}}_{y,y}^{C})+({\bar{Q}}_{x,x}^{C}+{\bar{Q}}_{y,y}^{C})+q]\delta wdxdy=0$$

Formulas (A22) and (A29) are built analogously, but these equations have two variables w,φ for the S-FDST and only one variable w for the CPT. As in the FSDT and the S-FSDT, the bending components of transverse forces ${\hat{Q}}_{x}^{C}$ and ${\hat{Q}}_{y}^{C}$ are linearly dependent on the variable w, whereas the membrane components ${\bar{Q}}_{x}^{C}$ and ${\bar{Q}}_{y}^{C}$ are nonlinearly dependent on the variables F,w.

### Appendix A.2. Nonlinear Problem of Distributions of Transverse Shear Forces in the Square Plate Subject Simultaneously to Compression and Transverse Loading

A square isotropic plate fixed along all edges and simultaneously subject to the compression *p* along the *x* axis direction and the constant transverse load *q* (Figure 1) was analyzed. The plate characterized by the length *a*, the thickness *h*, and the material constants Young’s modulus *E* and Poisson’s ratio ν was investigated in an elastic range only. The problem was solved within the first-order nonlinear approximation.

#### Appendix A.2.1. Equation of Inseparability of Deformations

The equation of inseparability of deformations (A9) is identical for the three theories under investigation.

The deflection of the plate fixed along all edges within the first-order approximation was approximated with the following function [43]:

(A30) $$w=W\sin^{2}\frac{\pi x}{a}\sin^{2}\frac{\pi y}{a}$$

which fulfilled the following boundary conditions:

(A31) $$w(x=0)=w(x=a)=w(y=0)=w(y=a)=0\;w_{,x}(x=0)=w_{,x}(x=a)=w_{,y}(y=0)=w_{,y}(y=a)=0$$

After submission of (A30) into (A9), a function of Airy forces *F* was defined:

(A32) $$\begin{array}{l} F=-\frac{py^{2}}{2}+EW^{2}(\frac{1}{32}\cos\frac{2\pi x}{a}+\frac{1}{32}\cos\frac{2\pi y}{a}-\frac{1}{512}\cos\frac{4\pi x}{a}-\frac{1}{512}\cos\frac{4\pi y}{a}\\ +\frac{1}{800}\cos\frac{4\pi x}{a}\cos\frac{2\pi y}{a}+\frac{1}{800}\cos\frac{2\pi x}{a}\cos\frac{4\pi y}{a}-\frac{1}{64}\cos\frac{2\pi x}{a}\cos\frac{2\pi y}{a}) \end{array}$$

from the above and from (A8) it follows

(A33) $$\begin{array}{l} N_{x}=F_{,yy}=-p+EW^{2}{\left(\frac{\pi }{a}\right)}^{2}[-\frac{1}{8}\cos\frac{2\pi y}{a}+\frac{1}{32}\cos\frac{4\pi y}{a}-\frac{1}{200}\cos\frac{4\pi x}{a}\cos\frac{2\pi y}{a}\\ -\frac{1}{50}\cos\frac{2\pi x}{a}\cos\frac{4\pi y}{a}+\frac{1}{16}\cos\frac{2\pi x}{a}\cos\frac{2\pi y}{a}]\\ N_{y}=F_{,xx}=EW^{2}{\left(\frac{\pi }{a}\right)}^{2}[-\frac{1}{8}\cos\frac{2\pi x}{a}+\frac{1}{32}\cos\frac{4\pi x}{a}-\frac{1}{50}\cos\frac{4\pi x}{a}\cos\frac{2\pi y}{a}\\ -\frac{1}{200}\cos\frac{2\pi x}{a}\cos\frac{4\pi y}{a}+\frac{1}{16}\cos\frac{2\pi x}{a}\cos\frac{2\pi y}{a}]\\ N_{xy}=-F_{,xy}=-EW^{2}{\left(\frac{\pi }{a}\right)}^{2}[\frac{1}{100}\sin\frac{4\pi x}{a}\sin\frac{2\pi y}{a}+\frac{1}{100}\sin\frac{2\pi x}{a}\sin\frac{4\pi y}{a}-\frac{1}{16}\sin\frac{2\pi x}{a}\sin\frac{2\pi y}{a}] \end{array}$$

Functions of forces (A33) fulfill the boundary conditions [30,33]:

(A34) $$u(x=0)=u(x=a)=const\;N_{xy}(x=0)=N_{xy}(x=a)=0\;v(y=0)=v(y=a)=const\;N_{xy}(y=0)=N_{xy}(y=a)=0$$

#### Appendix A.2.2. CPT-Solution to the Nonlinear Problem of Stability

In regard to the CPT, the Galerkin–Bubnov method was used to solve the nonlinear problem of stability (A25) or (A29) with respect to *w.* When the function of forces (A32) and the function of deflection *w* (A30) were introduced, a nonlinear equation of equilibrium of the square plate subject to simultaneous compression and the uniform transverse loading *q* was attained for the CPT within the first approximation [43]:

(A35) $$\frac{533\pi^{4}}{3200}\zeta^{3}-\frac{3\pi^{2}}{4}{\bar{p}}^{C}\zeta +\frac{2\pi^{4}}{3(1-\nu^{2})}\zeta ={\bar{q}}^{C}$$

where:

(A36) $${\bar{p}}^{C}=\frac{p^{C}a^{2}}{Eh^{2}},\;{\bar{q}}^{C}=\frac{q^{C}a^{4}}{Eh^{4}}\;\mathrm{and}\;\zeta =W/h$$

For Equation (A35), two particular cases can be considered, namely:

- ${\bar{p}}^{C}\neq 0$ and ${\bar{q}}^{C}=0$ (the plate subject to uniform compression, transverse loading neglected)

In this case, we have a nonlinear stability problem of the plate in compression and Equation (A35) is simplified to

(A37) $$\frac{533\pi^{4}}{800}\zeta^{3}+({\bar{p}}_{cr}^{C}-{\bar{p}}^{C})\zeta =0$$

where:

(A38) $${\bar{p}}_{cr}^{C}=\frac{p_{cr}^{C}a^{2}}{Eh^{2}}=\frac{8\pi^{2}}{9(1-\nu^{2})}\text{—}\mathrm{dimensionless}\;\mathrm{critical}\;\mathrm{stress}.$$

The critical stress, according to (A38), can be expressed as

(A39) $$p_{cr}^{C}=\frac{32D\pi^{2}}{3a^{2}h}=10.67\frac{D\pi^{2}}{a^{2}h}$$

The value of the critical stress for the first approximation was deviated by 1.6% with respect to the accurate value of 10.5 [43].

- ${\bar{p}}^{C}=0$ and ${\bar{q}}^{C}\neq 0$ (the plate subject to uniform transverse loading, compression neglected)

In this case, we have a nonlinear problem of deflection of the thin plate, accompanied by an appearance of membrane forces (A33):

(A40) $$\frac{533\pi^{4}}{3200}\zeta^{3}+\frac{2\pi^{4}}{3(1-\nu^{2})}\zeta ={\bar{q}}^{C}$$

The determined dimensionless deflections ζ for the assumed value of transverse load from Equation (A40), within the first-order approximation, were lower by 3.5% with respect to the accurate solution [43].

The equivalent Kirchhoff transverse forces (A26), i.e., the bending components of transverse forces, when (A30) is considered, are expressed with the following relationships:

(A41) $$\begin{array}{l} {\hat{Q}}_{x}^{C}=M_{x,x}^{C}+2M_{xy,y}^{C}=2DW{\left(\frac{\pi }{a}\right)}^{3}\left(\sin\frac{2\pi x}{a}-(3-\nu )\sin\frac{2\pi x}{a}\cos\frac{2\pi y}{a}\right)\\ {\hat{Q}}_{y}^{C}=M_{y,y}^{C}+2M_{xy,x}^{C}=2DW{\left(\frac{\pi }{a}\right)}^{3}\left(\sin\frac{2\pi y}{a}-(3-\nu )\cos\frac{2\pi x}{a}\sin\frac{2\pi y}{a}\right) \end{array}$$

As one can easily notice in (A41), the bending components are linearly dependent on the deflection *W*.

When we take (A30) and (A33) into account, the membrane components (A27) are as follows:

(A42) $$\begin{array}{l} {\bar{Q}}_{x}^{C}=N_{x}w_{,x}+N_{xy}w_{,y}=-EW^{3}h{\left(\frac{\pi }{a}\right)}^{3}[(\frac{1}{8}\cos\frac{2\pi y}{a}-\frac{1}{32}\cos\frac{4\pi y}{a}+\frac{1}{200}\cos\frac{4\pi x}{a}\cos\frac{2\pi y}{a}+\\ \frac{1}{50}\cos\frac{2\pi x}{a}\cos\frac{4\pi y}{a}-\frac{1}{16}\cos\frac{2\pi x}{a}\cos\frac{2\pi y}{a})\sin\frac{2\pi x}{a}(\frac{1}{2}-\frac{1}{2}\cos\frac{2\pi y}{a})+\\ (\frac{1}{100}\sin\frac{4\pi x}{a}\sin\frac{2\pi y}{a}+\frac{1}{100}\sin\frac{2\pi x}{a}\sin\frac{4\pi y}{a}-\frac{1}{16}\sin\frac{2\pi x}{a}\sin\frac{2\pi y}{a})(\frac{1}{2}-\frac{1}{2}\cos\frac{2\pi x}{a})\sin\frac{2\pi y}{a}]-\\ p^{C}Wh\left(\frac{\pi }{a}\right)\sin\frac{2\pi x}{a}(\frac{1}{2}-\frac{1}{2}\cos\frac{2\pi y}{a})\\ {\bar{Q}}_{y}^{C}=N_{y}w_{,y}+N_{xy}w_{,x}=-EW^{3}h{\left(\frac{\pi }{a}\right)}^{3}[(\frac{1}{8}\cos\frac{2\pi x}{a}-\frac{1}{32}\cos\frac{4\pi x}{a}+\frac{1}{50}\cos\frac{4\pi x}{a}\cos\frac{2\pi y}{a}+\\ \frac{1}{200}\cos\frac{2\pi x}{a}\cos\frac{4\pi y}{a}-\frac{1}{16}\cos\frac{2\pi x}{a}\cos\frac{2\pi y}{a})(\frac{1}{2}-\frac{1}{2}\cos\frac{2\pi x}{a})\sin\frac{2\pi y}{a}+\\ \left(\frac{1}{100}\sin\frac{4\pi x}{a}\sin\frac{2\pi y}{a}+\frac{1}{100}\sin\frac{2\pi x}{a}\sin\frac{4\pi y}{a}-\frac{1}{16}\sin\frac{2\pi x}{a}\sin\frac{2\pi y}{a})\sin\frac{2\pi x}{a}(\frac{1}{2}-\frac{1}{2}\cos\frac{2\pi y}{a})\right] \end{array}$$

The membrane components (A42) are nonlinearly dependent on the deflection *W*, or strictly speaking, on *W* raised to the third power. The last term in the first equation is linearly dependent on *W* for the case of compression, whereas when we have the transverse load *q* only, this term becomes zero.

The total components of transverse forces, i.e., total equivalent Kirchhoff forces, are expressed with Formula (A28).

#### Appendix A.2.3. S-FSDT-Solution to the Nonlinear Problem of Stability

A solution to system of Equation (A18) for the S-FSDT is predicted as (A30) with respect to the variable *w* and for the variable φ in the form

(A43) $$\varphi =\Phi^{S}\sin^{2}\frac{\pi x}{a}\sin^{2}\frac{\pi y}{a}$$

When variables (A30) and (A43) are substituted into (A16)–(A19) into the second equation, the following relationship is obtained:

(A44) $$\Phi^{S}=\frac{W}{1+\eta }=\alpha W$$

where: $\eta =\frac{2\pi^{2}}{3(1-\nu )k^{2}}{\left(\frac{h}{a}\right)}^{2}$, and, moreover, the reduction factor

(A45) α=1/(1+η)

When (A44) is taken into account, the first nonlinear Equation (A18) is solved with the Galerkin–Bubnov method to obtain

(A46) $$\frac{533\pi^{4}}{3200}\zeta^{3}-\frac{3\pi^{2}}{4}{\bar{p}}^{S}\zeta +\frac{2\pi^{4}}{3(1-\nu^{2})}\alpha \zeta ={\bar{q}}^{S}$$

where:

(A47) $${\bar{p}}^{S}=\frac{p^{S}a^{2}}{Eh^{2}}{\bar{q}}^{S}=\frac{q^{S}a^{4}}{Eh^{4}}$$

Like for the CPT, two particular cases were considered for (A46), namely:

- ${\bar{p}}^{S}\neq 0$*and*${\bar{q}}^{S}=0$*(the plate subject to uniform compression, transverse loading neglected)*

In this case, (A46) is simplified to

(A48) $$\frac{533\pi^{4}}{800}\zeta^{3}+({\bar{p}}_{cr}^{S}-{\bar{p}}^{S})\zeta =0$$

where the dimensionless critical load, when (A39) is accounted for, can be written as

(A49) $${\bar{p}}_{cr}^{S}=\frac{p_{cr}^{S}a^{2}}{Eh^{2}}=\frac{8\pi^{2}}{9(1-\nu^{2})}\alpha ={\bar{p}}_{cr}^{C}\alpha$$

- ${\bar{p}}^{S}=0$ *and* ${\bar{q}}^{S}\neq 0$ *(the plate subject to uniform transverse loading, compression neglected)*

In this case, we have a nonlinear problem of deflection of the plate, accompanied by an appearance of membrane forces:

(A50) $$\frac{533\pi^{4}}{3200}\zeta^{3}+\frac{2\pi^{4}}{3(1-\nu^{2})}\alpha \zeta ={\bar{q}}^{S}$$

The bending components of transverse forces for the S-FSDT, when (A41) and (A44) were accounted for, are expressed with the formula

(A51) $${\hat{Q}}_{x}^{S}={\hat{Q}}_{x}^{C}\alpha \;{\hat{Q}}_{y}^{S}={\hat{Q}}_{y}^{C}\alpha$$

Similarly as for the CPT, the bending components are linearly dependent on the deflection *W*.

The membrane components of transverse forces for the S-FSDT, according to (A20) and (A27) and when (A42) was considered, have the form

(A52) $${\bar{Q}}_{x}^{S}={\bar{Q}}_{x}^{C}\;{\bar{Q}}_{y}^{S}={\bar{Q}}_{y}^{C}$$

whereas the components of total transverse forces–(A21), correspondingly.

#### Appendix A.2.4. FSDT-Solution to the Nonlinear Problem of Stability

For the FSDT, a solution to the system of the last three Equations (A10) was predicted like (A30) with respect to the variable *w* and for the variables $\psi_{x},\psi_{y}$ in the form

(A53) $$\begin{array}{l} \psi_{x}=\Psi_{x}^{F}\sin\frac{2\pi x}{a}\sin^{2}\frac{\pi y}{a}\\ \psi_{y}=\Psi_{y}^{F}\sin^{2}\frac{\pi x}{a}\sin\frac{2\pi y}{a} \end{array}$$

When variables (A30), (A53), and (A45) were substituted, the following relationships were obtained:

(A54) $$\Psi_{x}^{F}=\Psi_{y}^{F}=W\alpha {\left(\frac{\pi }{a}\right)}^{2}$$

(A55) $$\frac{533\pi^{4}}{3200}\zeta^{3}-\frac{3\pi^{2}}{4}{\bar{p}}^{F}\zeta +\frac{2\pi^{4}}{3(1-\nu^{2})}\alpha \zeta ={\bar{q}}^{F}$$

where:

(A56) $${\bar{p}}^{F}=\frac{p^{F}a^{2}}{Eh^{2}},\;{\bar{q}}^{F}=\frac{q^{F}a^{4}}{Eh^{4}}$$

Like for the CPT and S-FSDT, two particular cases were considered for (A55), namely:

- ${\bar{p}}^{F}\neq 0$*and*${\bar{q}}^{F}=0$*(the plate subject to uniform compression, transverse loading neglected)*

In this case, (A55) simplifies to

(A57) $$\frac{533\pi^{4}}{800}\zeta^{3}+({\bar{p}}_{cr}^{F}-{\bar{p}}^{F})\zeta =0$$

where the dimensionless critical stress, when (A49) and (A39) were considered, takes the form

(A58) $${\bar{p}}_{cr}^{F}=\frac{p_{cr}^{F}a^{2}}{Eh^{2}}=\frac{8\pi^{2}}{9(1-\nu^{2})}\alpha ={\bar{p}}_{cr}^{S}={\bar{p}}_{cr}^{C}\alpha$$

- ${\bar{p}}^{F}=0$ *and* ${\bar{q}}^{F}\neq 0$ *(the plate subject to uniform transverse loading, compression neglected)*

The nonlinear problem of deflection of the thin plate, accompanied by an appearance of membrane components, was expressed as

(A59) $$\frac{533\pi^{4}}{3200}\zeta^{3}+\frac{2\pi^{4}}{3(1-\nu^{2})}\alpha \zeta ={\bar{q}}^{F}$$

When (A59) and (A50) are compared, one can see that ${\bar{q}}^{S}={\bar{q}}^{F}$.

The bending components of transverse forces according to (A11) are equal to

(A60) $$\begin{array}{l} {\hat{Q}}_{x}^{F}=M_{x,x}^{F}+M_{xy,y}^{F}=2DW{\left(\frac{\pi }{a}\right)}^{3}\left(\sin\frac{2\pi x}{a}-2\sin\frac{2\pi x}{a}\cos\frac{2\pi y}{a}\right)\alpha \\ {\hat{Q}}_{y}^{F}=M_{y,y}^{F}+M_{xy,x}^{F}=2DW{\left(\frac{\pi }{a}\right)}^{3}\left(\sin\frac{2\pi y}{a}-2\cos\frac{2\pi x}{a}\sin\frac{2\pi y}{a}\right)\alpha \end{array}$$

Like for the CPT and S-FSDT, the bending components are linearly dependent on the deflection *W*. Comparing (A60) and (A51), we can see that there is a factor equal to 2 at the second term in the bracket for the FSDT, whereas for the S-FSDT, the factor is (3-ν).

The membrane components of transverse forces (A12) for the FSDT are identical as in the case of the S-FSDT and the CPT (A52):

(A61) $${\bar{Q}}_{x}^{F}={\bar{Q}}_{x}^{S}={\bar{Q}}_{x}^{C}\;{\bar{Q}}_{y}^{F}={\bar{Q}}_{y}^{S}={\bar{Q}}_{y}^{C}$$

whereas the components of total transverse forces are the same and are given in (A13), (A21), and (A28), respectively.
